# Delayed Tracheal Wall Injury Following Percutaneous Tracheostomy Tube Exchange Resulting in Pneumomediastinum: A Case Report

**DOI:** 10.7759/cureus.98159

**Published:** 2025-11-30

**Authors:** Avinash Sharma, Sashank Mavuduru, Apoorva Magu, Ajit Thakur, Anshuman Panda

**Affiliations:** 1 Critical Care Medicine, Asian Institute of Medical Sciences, Faridabad, IND

**Keywords:** difficult airway management, fiberoptic bronchoscopy, percutaneous tracheostomy, tension pneumomediastinum, tracheal injuries

## Abstract

A tracheal wall injury is a rare but serious complication of percutaneous dilatational tracheostomy (PDT). Most tears occur during the index procedure; delayed posterior membranous rupture after routine tube exchange is uncommon and may present subtly until significant airway or mediastinal compromise develops.

We report a 66-year-old woman who developed rapidly progressive subcutaneous emphysema, hypoxia, and early tension physiology shortly after a routine tracheostomy tube exchange performed eight days after an initially uncomplicated PDT. Imaging revealed extensive pneumomediastinum, and bronchoscopy identified a longitudinal posterior tracheal tear 1-2 cm above the carina. Management required immediate decompression, temporary orotracheal intubation, and placement of an adjustable long-flange tracheostomy tube beyond the defect, followed by definitive endoluminal stenting. This strategy stabilized the airway and prevented further mediastinal air leak. Delayed tracheal injury following tracheostomy tube exchange is an under-recognized cause of acute subcutaneous emphysema and respiratory deterioration. Early suspicion, bronchoscopic confirmation, rapid decompression when tension physiology is present, and individualized airway reconstruction, including distal tube positioning or stenting, are critical to preventing catastrophic outcomes.

## Introduction

Percutaneous dilatational tracheostomy (PDT) is a commonly performed bedside procedure in intensive care units to facilitate prolonged mechanical ventilation and airway clearance. Although generally safe, PDT is associated with rare but potentially life-threatening complications such as tracheal wall injury, bleeding, or pneumothorax. The reported incidence of tracheal tears following PDT ranges between approximately 0.1% and 1% in published series [1]. Tracheal tears may present immediately or be delayed, often occurring after subsequent airway manipulation, such as tube exchange, rather than at the time of the initial procedure [2]. The posterior membranous tracheal wall is particularly vulnerable because it lacks cartilaginous support and is therefore more susceptible to shearing and pressure-related injury during dilation or manipulation [3]. Recently, advancements in procedural technique, including ultrasound-assisted PDT and improved bronchoscopic protocols, have been proposed to reduce airway trauma and enhance patient safety [4-6].

## Case presentation

A 66-year-old woman with a history of right cerebellar infarct with hemorrhagic transformation underwent decompressive craniectomy and was admitted to the intensive care unit for postoperative ventilatory support. She was maintained on continuous positive airway pressure (CPAP) mode for weaning, ventilated with a pressure support of 12 cmH₂O, positive end-expiratory pressure (PEEP) of 6 cmH₂O, and fraction of inspired oxygen (FiO₂) of 0.4. As prolonged ventilation was anticipated, a percutaneous tracheostomy was performed on day five using the Seldinger technique under bronchoscopic guidance. The procedure was performed by an experienced intensivist (>200 PDTs) using a standard 7.5 mm cuffed tracheostomy tube, with cuff pressures maintained between 22 and 26 cmH₂O. No procedural difficulty or mucosal injury was observed at the time.

On post-tracheostomy day eight, the tracheostomy tube was electively changed due to suspected internal blockage from secretions. A guidewire-assisted exchange was performed under aseptic conditions. The wire advanced without any noticeable resistance, and the procedure appeared uneventful. Immediately after the exchange, the patient remained on the same ventilator settings (pressure support 12, PEEP 6, FiO₂ 0.4) with stable parameters for the first 10-15 minutes. Shortly thereafter, she developed rapidly progressive subcutaneous emphysema over the neck and chest, with a fall in oxygen saturation to 85% despite increasing FiO₂ to 0.8. Peak airway pressures increased from 22 to 32 cmH₂O, and rapid-onset hemodynamic instability ensued. Palpable crepitus extended to the face, chest, and upper limbs, raising high suspicion of a tracheal injury with air tracking into the mediastinum.

**Figure 1:**
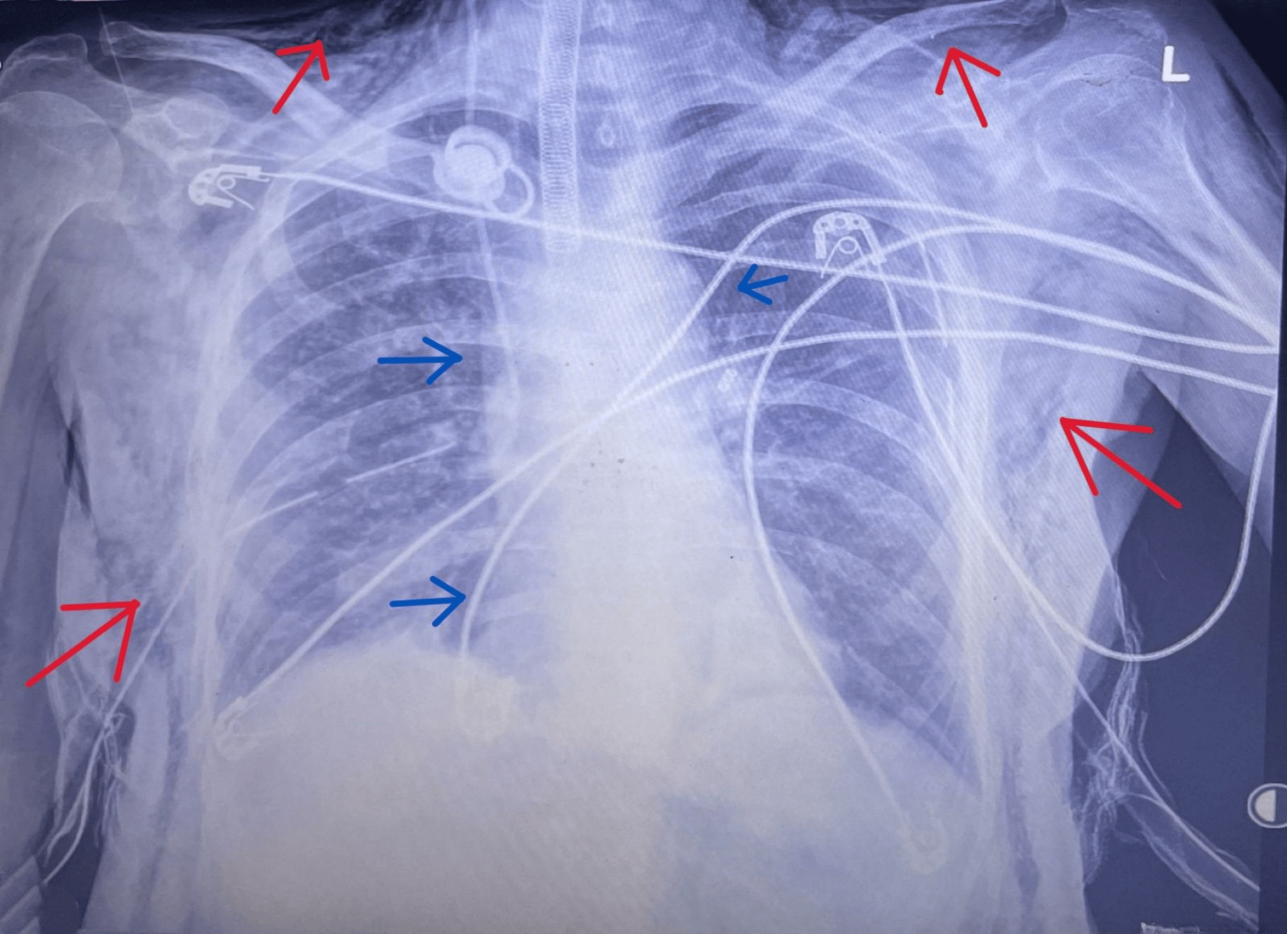
Chest radiograph showing pneumomediastinum and extensive subcutaneous emphysema Portable anteroposterior chest radiograph demonstrating extensive subcutaneous emphysema of the neck and chest (red arrows) and mediastinal air outlining the cardiac borders (blue arrows), consistent with pneumomediastinum. Bilateral intercostal drains are visible. The radiograph highlights the degree of air tracking and supports suspicion of tracheal injury.

A portable chest radiograph demonstrated extensive subcutaneous emphysema with clearly visible mediastinal air, consistent with pneumomediastinum, without pneumothorax, and with the tracheostomy tube appearing in a satisfactory position (Figure 1). This pattern of diffuse subcutaneous emphysema without pneumothorax is more characteristic of delayed or contained tracheal tears than of immediate post-procedural ruptures.

**Figure 2:**
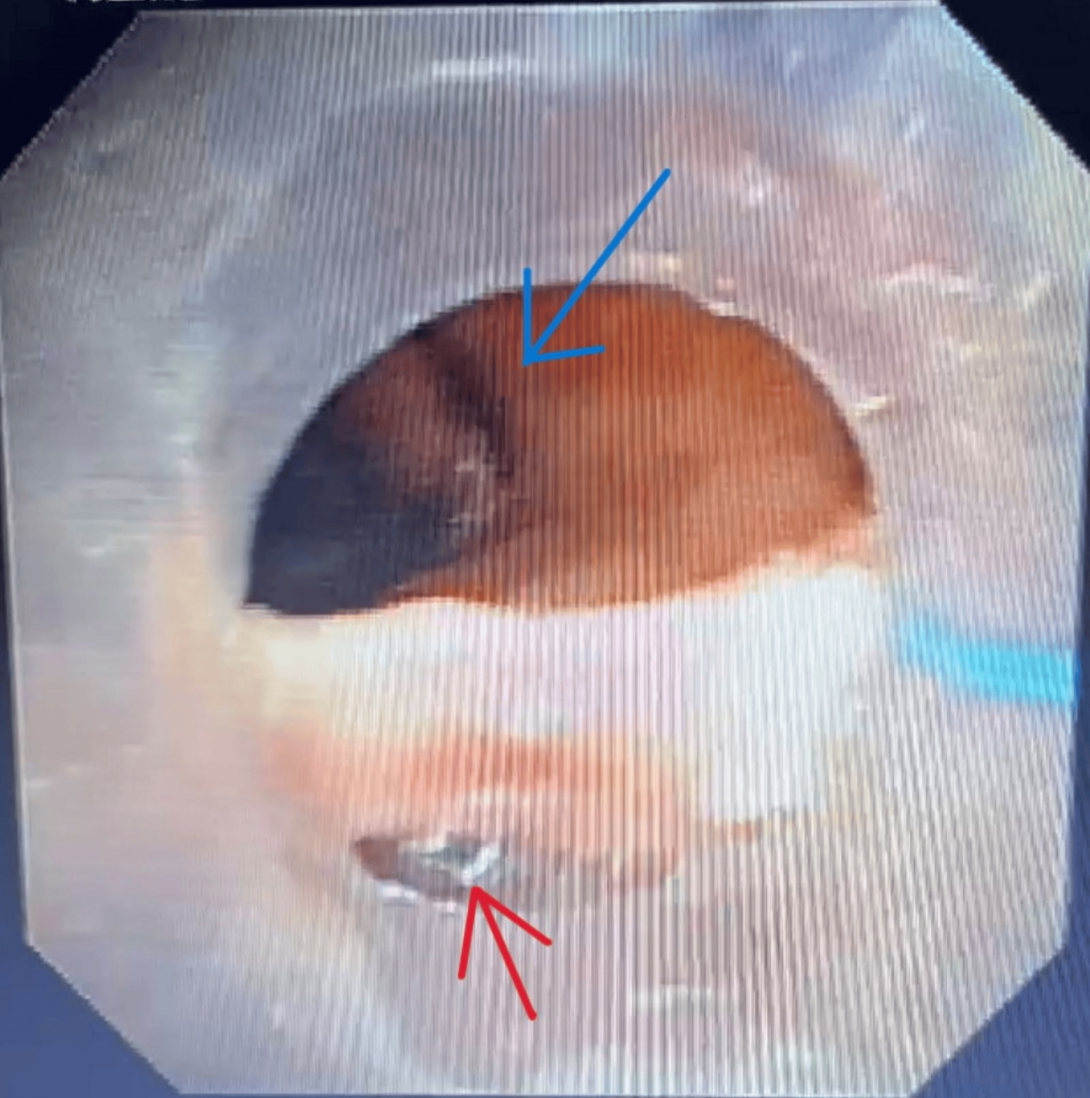
Bronchoscopic visualization of the posterior membranous tracheal tear Flexible bronchoscopy revealing a longitudinal posterior membranous tracheal tear (red arrow) located approximately 1–2 cm above the carina (blue arrow). The defect appears without active bleeding, consistent with a partial-thickness tear. This finding confirmed the airway source of the patient’s subcutaneous emphysema and pneumomediastinum.

Flexible bronchoscopy was performed and revealed a longitudinal 1-2 cm tear in the posterior membranous tracheal wall approximately 1-2 cm above the carina (Figure 2). The tear had smooth, non-bleeding margins, suggesting that the mucosal injury was likely older and had expanded or dehisced during the tube exchange rather than representing a fresh traumatic perforation. Upper gastrointestinal endoscopy ruled out esophageal injury.

**Figure 3:**
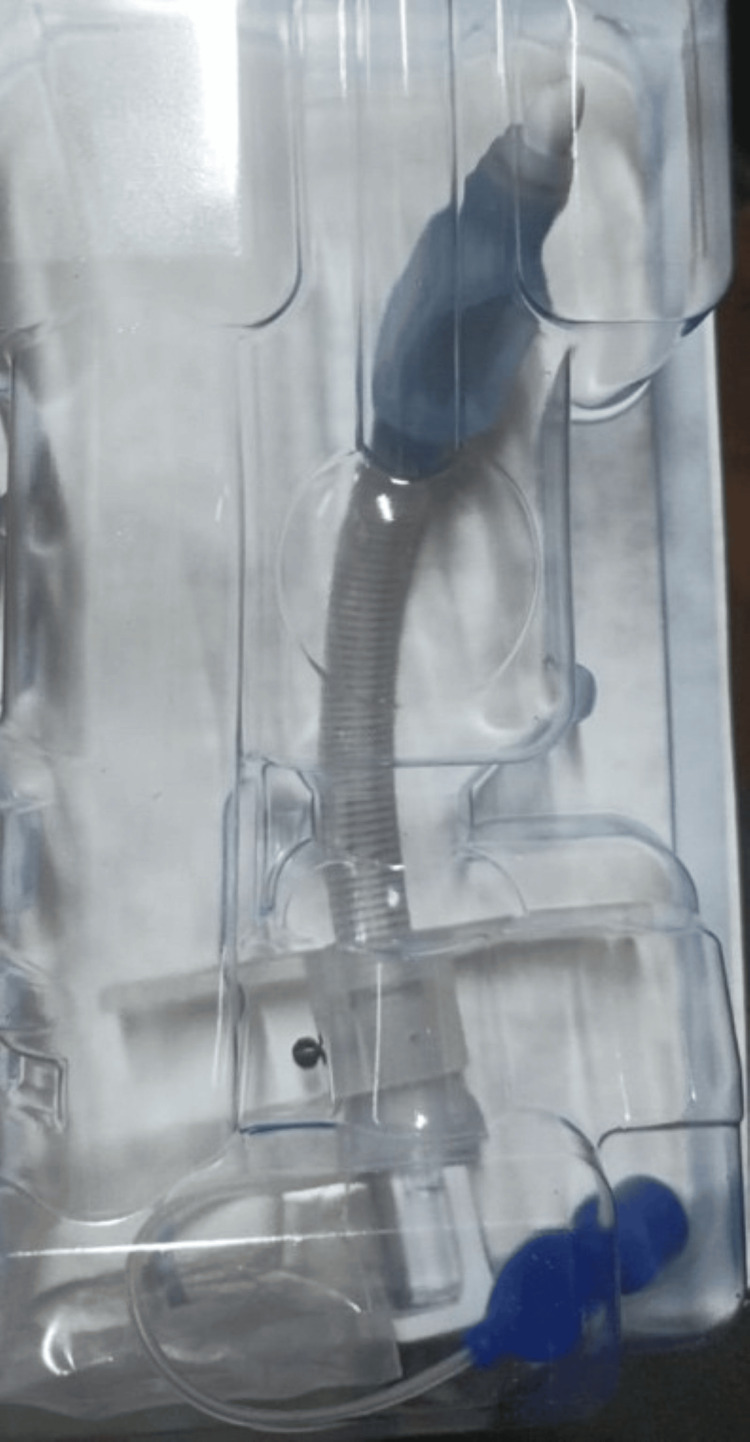
Adjustable long-flange tracheostomy tube used for airway stabilization Image of the adjustable, reinforced long-flange tracheostomy tube used to bypass the posterior tracheal wall defect. The extended distal length allowed secure placement beyond the membranous tear, reducing airway pressure on the injured segment and minimizing further air leak.

The patient was managed with low-tidal volume (4-6 mL/kg) and high-rate ventilation to minimize airflow across the defect. Bilateral intercostal drains were inserted to decompress the mediastinum and mitigate early tension physiology. The tracheostomy tube was removed, the stoma was strapped, and the airway was temporarily secured by orotracheal intubation. A size 8 reinforced adjustable long-flange tracheostomy tube (Portex®) was then inserted under bronchoscopic guidance, ensuring placement distal to the tracheal defect to prevent further leakage (Figure 3).

**Figure 4:**
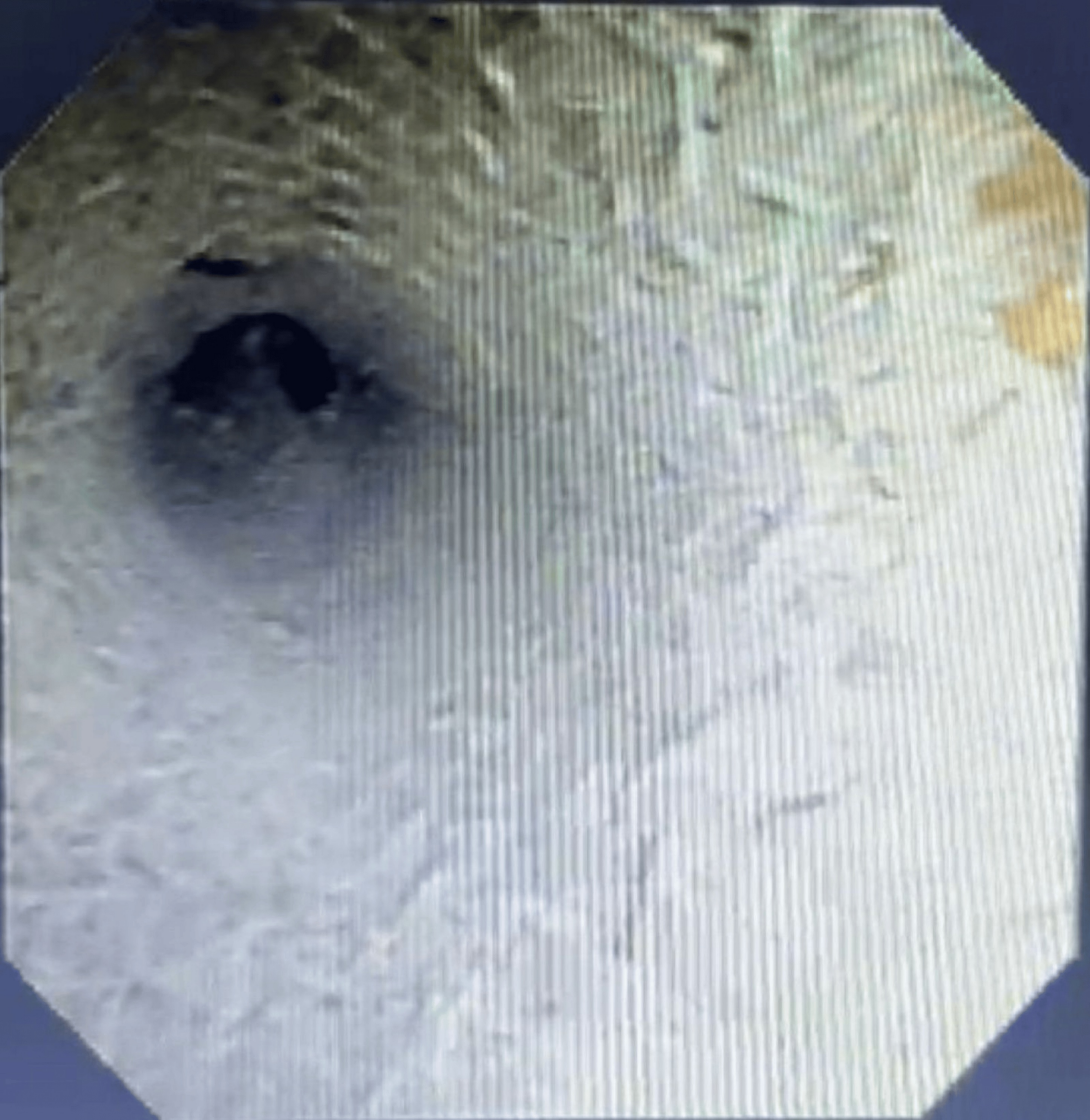
Bronchoscopic view following tracheal stent placement Bronchoscopic image showing deployment of an endoluminal tracheal stent used to stabilize the posterior wall tear. The stent provides internal scaffolding to minimize dynamic airway collapse and promotes controlled healing of the defect.

**Figure 5:**
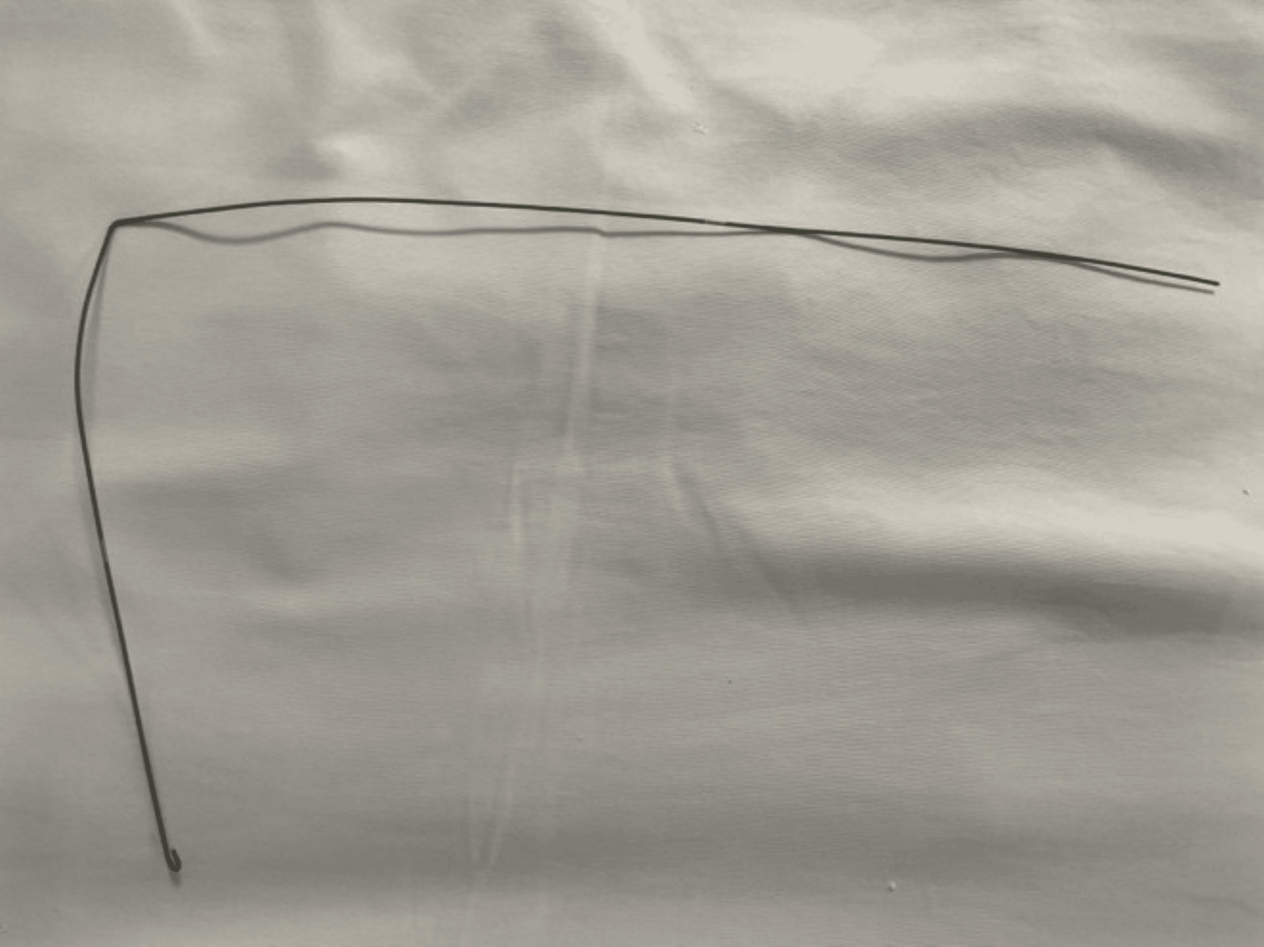
Metallic guidewire used during tracheostomy tube exchange Resistance encountered while advancing the tracheostomy tube over the guidewire was later correlated with the posterior tracheal wall mucosal defect observed on bronchoscopy. Although no immediate trauma was noted during the procedure, subsequent progression of the lesion supports a possible iatrogenic mechanism related to this guidewire passage.

Once stabilized, the patient underwent endoscopic placement of a tracheal stent to maintain luminal integrity and support mucosal healing (Figure 4). Review of equipment confirmed that the guidewire used during the exchange (Figure 5) was the most likely cause of the original mucosal injury, consistent with the tear’s orientation and delayed clinical manifestation.

The patient’s oxygenation and hemodynamic status improved promptly following these interventions. She remained stable and continued to recover with multidisciplinary care.

## Discussion

PDT is widely used in the intensive care setting due to its practicality and safety profile; however, iatrogenic tracheal injuries, although rare (0.1-1%), can result in significant morbidity and mortality. The posterior membranous wall remains the most vulnerable region due to its lack of cartilaginous reinforcement and greater susceptibility to mechanical or pressure trauma during procedural manipulation [1,2].

Clinically, rapidly progressive subcutaneous emphysema accompanied by hypoxia or rising airway pressures should immediately raise concern for an airway breach. While chest radiography is useful for identifying pneumomediastinum (Figure 1), bronchoscopy remains the diagnostic gold standard because it allows direct visualization of the tear location, orientation, and extent, facilitating management planning [3].

Mechanical factors, such as excessive guidewire advancement, resistance during tube exchange, over-dilatation, or unrecognized cuff overinflation, have been implicated in tracheal injuries [2,4]. In this patient, resistance encountered during tube exchange likely resulted in mucosal trauma from the guidewire (Figure 5), consistent with previously reported mechanisms [2]. The delayed presentation on post-tracheostomy day eight is clinically notable, as most injuries typically manifest within the first 24-48 hours [3], although guidewire-related trauma is a recognized mechanism of delayed tracheal rupture [5]. This case emphasizes that even an initially uneventful bronchoscopically guided PDT does not exclude later complications, particularly following airway manipulation.

Management strategies depend on tear size, anatomical location, and clinical severity. Conservative management may be appropriate in hemodynamically stable patients with partial-thickness tears, whereas cases with evidence of respiratory compromise or tension physiology require immediate intervention. Urgent airway securing using endotracheal intubation or careful tube repositioning is recommended in unstable cases in accordance with current airway safety principles [6]. In this patient, emergency decompression with bilateral intercostal drains resulted in rapid hemodynamic improvement [7]. Airway stabilization was subsequently achieved by placing an adjustable long-flange tracheostomy tube distal to the injury (Figure 3), reducing pressure at the tear site and facilitating healing [8]. Definitive endoluminal stenting (Figure 4) further supported airway integrity and promoted mucosal recovery [8,9].

The patient remained clinically stable at the time of hospital discharge; however, long-term follow-up could not be completed, as she did not return for scheduled outpatient bronchoscopic reassessment. The tracheal stent remained in situ at discharge with planned re-evaluation for potential removal upon confirmation of mucosal healing. The absence of post-discharge follow-up limits confirmation of long-term airway recovery and is acknowledged as a limitation of this report [10].

Recent evidence highlights the importance of multidisciplinary collaboration, typically involving intensivists, pulmonologists, and thoracic surgeons, in optimizing outcomes in airway trauma [11]. Additionally, ultrasound-assisted PDT and the availability of newer airway stenting technologies with improved design are emerging strategies aimed at reducing complication risk [1,8]. This case reinforces the need for vigilance beyond the immediate post-procedural period and supports physiologically guided interventions to manage delayed airway injury effectively. Future practice may benefit from structured preventive measures such as resistance-alert protocols during tube exchange, continuous cuff-pressure monitoring, real-time airway visualization, and a low threshold for early bronchoscopy in cases of subcutaneous emphysema [2-4].

## Conclusions

Iatrogenic tracheal injury following percutaneous tracheostomy or tube exchange, though rare, can result in life-threatening complications such as tension pneumomediastinum. This case underscores the importance of early recognition of warning signs, bronchoscopic localization of injury, and timely multidisciplinary intervention to prevent catastrophic outcomes. Particular attention should be given to delayed airway complications following seemingly uneventful PDT, especially after subsequent tube manipulation. Preventive strategies should include real-time airway visualization during tube exchanges, gentle guidewire handling, continuous cuff pressure monitoring, and early bronchoscopy when subcutaneous emphysema is detected. Although the patient was clinically stable at discharge, long-term confirmation of airway healing could not be established due to a lack of post-discharge follow-up.
